# The Invasive Blue Crab *Callinectes sapidus* as a Model for Assessing Sub-Lethal Effects of Polyvinyl Alcohol

**DOI:** 10.3390/toxics14050358

**Published:** 2026-04-24

**Authors:** Alessandra Maganza, Giorgia Zicarelli, Giuseppe Esposito, Annalisa Cotugno, Alice Gabetti, Camilla Mossotto, Alessia Merialdi, Francesca Provenza, Serena Anselmi, Marzia Pezzolato, Elena Bozzetta, Monia Renzi, Marino Prearo, Caterina Faggio, Edoardo Turolla, Antonia Concetta Elia, Paolo Pastorino

**Affiliations:** 1 Istituto Zooprofilattico Sperimentale del Piemonte, Liguria e Valle d’Aosta, Via Bologna 148, 10154 Turin, Italy; alessandra.maganza@izsplv.it (A.M.); giorgia.zicarelli@izsplv.it (G.Z.); annalisa.cotugno@izsplv.it (A.C.); alice.gabetti@izsplv.it (A.G.); camilla.mossotto@izsplv.it (C.M.); alessia.merialdi@izsplv.it (A.M.); marzia.pezzolato@izsplv.it (M.P.); elena.bozzetta@izsplv.it (E.B.); marino.prearo@izsplv.it (M.P.); 2 Department of Chemistry, Biology and Biotechnology, University of Perugia, Via Elce di Sotto 8, 06123 Perugia, Italy; antonia.elia@unipg.it; 3 Bioscience Research Center, via Aurelia Vecchia 32, 58015 Orbetello, Italy; francesca.provenza@bsrc.it (F.P.); serena.anselmi@bsrc.it (S.A.); 4 Department of Life Sciences, University of Trieste, Via L. Giorgieri 10, 34127 Trieste, Italy; mrenzi@units.it; 5 Department of Chemical, Biological, Pharmaceutical and Environmental Sciences, University of Messina, 98166 Messina, Italy; cfaggio@unime.it; 6 Department of Eco-Sustainable Marine Biotechnology, Stazione Zoologica Anton Dohrn, 80121 Naples, Italy; 7 Istituto Delta—Ecologia Applicata, 44124 Ferrara, Italy; veliger@istitutodelta.it

**Keywords:** aquatic pollution, emerging contaminants, oxidative stress biomarkers, water-soluble polymer

## Abstract

Polyvinyl alcohols (PVAs) are synthetic, water-soluble polymers widely used in industrial, medical, and personal care products. Their slow biodegradation raises concerns about potential impacts on marine ecosystems. This study examined how PVA exposure affects the blue crab *Callinectes sapidus*, an invasive species in the Mediterranean Sea. Crabs were exposed to three PVA concentrations (0.5, 5, and 25 mg L^−1^) along with a control group, for periods of 10 and 20 days. Oxidative stress was assessed by measuring antioxidant enzyme activities, including superoxide dismutase (SOD), glutathione S-transferase (GST), glutathione peroxidase (GPx), and lipid peroxidation levels in muscle, gill, and hepatopancreas. Cell viability in the hemolymph and hepatopancreas was also evaluated. The results showed that hepatopancreas cells were more sensitive than hemolymph cells. Oxidative stress increased with exposure time and concentration, as indicated by elevated antioxidant enzyme activity and lipid peroxidation. After 10 days, early detoxification responses were observed, while after 20 days of exposure, clear dose- and time-dependent trends were evident, highlighting an intensification of physiological dysfunctions with increasing PVA concentrations and prolonged exposure duration. The histopathological observations showed limited alterations in muscle and hepatopancreas tissue but evident structural changes in gill tissues, particularly after prolonged exposure. The findings reveal a concentration- and time-dependent biological response to PVA, highlighting physiological changes at higher exposure levels and the need for further research on environmental consequences.

## 1. Introduction

Plastic materials are considered one of the most important technological innovations of the 20th century, valued for their low cost, excellent mechanical performance, lightweight nature, and long-term durability [1]. Nevertheless, their extensive production and widespread use have turned them into one of the greatest threats to both the environment and public health. As a result, plastic pollution has become a major focus of concern, leading to a sharp rise in scientific publications on the topic [2]. As public awareness of plastic consumption and its environmental impact continue to grow, alternatives perceived as more sustainable and less harmful have gained popularity and are increasingly adopted by consumers [3,4,5].

Among these materials are water-soluble polymers (WSPs), often referred to as “liquid plastics”. These are substances capable of dissolving in water under specific pH or temperature conditions, but which may become insoluble if those conditions change. As a result, they can influence the viscosity of aqueous solutions and can be altered through dispersion or dissolution in water to create gelled, stabilized, concentrated, or emulsified formulations [6].

Polyvinyl alcohols (PVAs) represent a specific category of synthetic water-soluble biopolymers, typically obtained through the saponification of polyvinyl acetate [7].

Introduced in the latter half of the 20th century, PVAs are widely used in the production of textile and industrial fibers, adhesives, binders, water-soluble films for packaging materials, and in detergent pods [8,9]. Moreover, they are used in medical, pharmaceutical and personal care products, such as contact lenses, artificial tear drops, surgical sponges, and various cosmetics including facial masks, creams, and hair treatments [9,10,11]. Their chemical structure consists of a chain of carbon atoms with vinyl acetate ester groups replaced by hydroxyl groups, resulting in the repeating unit ((OH–CH–CH_2_)n), where n denotes the average number of hydroxyl groups [12].

PVA-based products are among the most prevalent WSPs found in the environment, with more than 65,000 tons entering aquatic ecosystems globally each year. Although they are widely used, earlier research has shown that PVAs degrade slowly and only under specific environmental conditions [5,13]. Indeed, Suaria et al. [14] found that PVA made up around 1.2% of all floating particles larger than 700 µm in the Mediterranean Sea, underscoring the persistence of this polymer in the environment. Moreover, PVA, like other WSPs, is currently not registered under the European Union’s REACH (Registration, Evaluation, Authorisation and Restriction of Chemicals) regulation. Consequently, reliable data on its production volumes are lacking, and significant uncertainties remain regarding its environmental fate and potential impacts [15,16]. Although existing toxicological assessments have determined that PVA is safe for use in cosmetic formulations and does not pose a risk to human health [17], its environmental behavior and long-term effects are still insufficiently known.

The environmental toxicity of PVA has been investigated in different studies, primarily focusing on freshwater organisms. Acute toxicity assays, such as those conducted on *Daphnia magna* and *Artemia salina*, have generally indicated low toxicity of PVA [18,19]. Similarly, algal growth inhibition tests showed effects only at relatively high concentrations; however, these experiments were performed using PVA stabilized with silver nanoparticles. In this case, the observed EC_50_ values were comparable to those reported in studies conducted with silver nanoparticles alone, suggesting that the primary toxic effect was attributable to the nanoparticles rather than to PVA itself. Although algal growth inhibition tests are already considered chronic assays despite the relatively short exposure duration (96 h), information on prolonged chronic effects of PVA over longer timescales remains scarce in the literature [18].

At the chronic level, Nigro et al. [20] investigated behavioral alterations in swimming performance of *D. magna* and *Danio rerio* and the activity of monoamine oxidase (MAO), a neuroenzyme potentially involved in locomotion for 14 days. At the end of the exposure, no significant differences were detected between the tested concentrations and the control. In contrast, Zicarelli et al. [21] evaluated the embryotoxicity of PVA in *D. rerio* and *Xenopus laevis* after 96 h of exposure, analyzing mortality, hatching success, malformations, and heart rate. The most relevant effects included delayed hatching in *D. rerio*, the occurrence of malformations (such as edema, body deformities, pigmentation changes, and spine and tail alterations) in both species, and significant variations in heart rate (either increases or decreases) in both organisms across all tested chemicals.

More recently, Multisanti et al. [22] investigated the effects of PVA on *Mytilus galloprovincialis* over a 14-day exposure period, assessing oxidative stress biomarkers, cellular viability, and the ability to produce the byssal plaque. Their results revealed alterations in cell volume regulatory capacity, changes in superoxide dismutase activity, and increased levels of oxidatively modified proteins in gills. In addition, PVA exposure led to modifications in byssal plaque production. Finally, embryotoxicity tests conducted on the sea urchin *Paracentrotus lividus* showed that only some PVA polymers induced slight toxic effects [23].

Considering the increasing release of emerging contaminants into marine ecosystems, it is essential to investigate their effects on the health of aquatic organisms. Water-soluble polymers, and particularly PVA, represent a growing and still insufficiently explored class of pollutants with the potential to induce biological damage. While the ecological impacts of petrochemical-based micro and nanoplastics have been extensively investigated, information on the toxicity of WSPs remains limited, despite steadily increasing research interest. Although standardized ecotoxicological tests provide reproducible and comparable data, they may fail to capture the complexity of contaminant and biota interactions, especially under environmentally realistic exposure scenarios. In this context, sub-chronic and chronic approaches are particularly valuable, as they enable the detection of early and sublethal effects that may precede population-level impacts.

To address this knowledge gap, the present study employed the invasive marine blue crab (*Callinectes sapidus*) as a model organism. Native to the western Atlantic, *C. sapidus* is an euryhaline decapod crustacean that has become invasive in the Mediterranean Sea, where it poses a serious threat to local biodiversity, fisheries, and aquaculture [24,25]. It is listed among the 100 worst marine invasive species due to its ecological impact in the Mediterranean Sea [26,27]. Decapod crustaceans have also emerged as valuable models in biochemical, physiological, and ecological research, owing to several advantageous traits, such as well-developed endocrine systems, strong immune responses, ease of collection and cultivation, manageable size, clear individual characteristics, resilience to handling, high fecundity, short generation times, and adaptability to diverse environmental and nutritional conditions [28]. Within this group, marine crabs have long been used as model organisms in ecotoxicological research and as sentinels for assessing the impacts of toxic environmental contaminants [29]. In the Mediterranean Sea, the crab *Carcinus aestuarii* has been extensively employed in such studies [30,31,32,33,34,35]. However, increasing attention is now being directed toward the blue crab, which represents a priority concern in the Mediterranean Sea due to its substantial ecological and economic impacts.

*C. sapidus* is a well-studied and thoroughly characterized species [36,37,38], making it a suitable model for investigating the effects of sublethal pollutants under chronic or sub-chronic exposure scenarios [38,39,40]. The species has adapted to temperate environments and a wide range of estuarine salinities, owing to its strong osmoregulatory and thermal capabilities. Reproduction occurs in areas with low-to-intermediate salinity, after which the females migrate towards higher-salinity waters for egg incubation and larval release. Development comprises seven zoeal stages and one megalopal stage. The larvae develop in coastal waters and subsequently migrate back to estuaries, where they settle and grow in nursery habitats [37].

The aim of this study was to evaluate the sublethal effects of PVA exposure in *C. sapidus* through a multi-biomarker approach. Oxidative stress biomarkers were assessed by measuring the activities of superoxide dismutase (SOD), glutathione S-transferase (GST), and glutathione peroxidase (GPx), along with lipid peroxidation levels through malondialdehyde (MDA) in muscle, gill, and hepatopancreas tissues. Considering the role of hemolymph as a first line of defense and the hepatopancreas as a key detoxification organ in crustaceans, cell viability was assessed in both tissues to detect potential alterations [41]. Additionally, histological analyses were performed on all three tissues (muscle, gill, and hepatopancreas) to identify possible structural damages due to PVA exposure.

## 2. Materials and Methods

### 2.1. Crab Sampling and Maintenance

**Figure 1:**
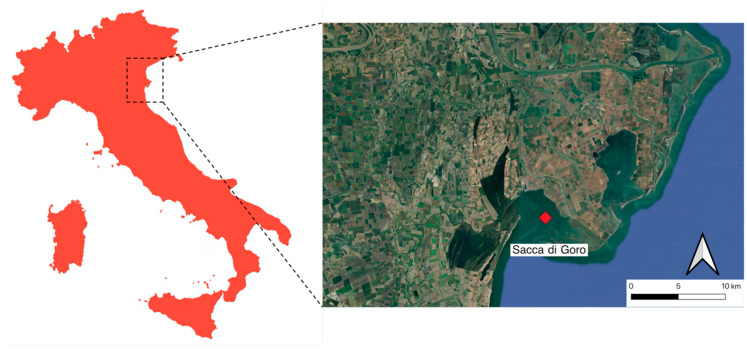
Sampling site in Sacca di Goro, Ferrara, Italy. Map created with QGIS Bratislava 3.40.

A single sampling session was conducted in June 2025 in Sacca di Goro, Italy (44°48′57″ N, 12°19′09″ E), shown in Figure 1. The site is a transitional zone; it is a lagoon that connects the Po Delta to the northern part of the Adriatic Sea, and its distinctive features have long been the subject of studies [42].

As crustaceans are not covered by Legislative Decree No. 26 of 4 March 2014 [43,44], authorization was not required. Moreover, the blue crab has been designated as an invasive alien species (IAS) under Regulation (EU) No. 1143/2014 [45] and Legislative Decree No. 230 of 15 December 2017 [46]. Handling practices were therefore carried out in accordance with regulations aimed at preventing the spread of IAS.

A total of 60 blue crabs (*Callinectes sapidus*) were sampled using fish-baited traps set at depths between 0.5 and 1.0 m [25,47]. The specimens were subsequently transported in refrigerated tanks to the Regional Reference Centre for Aquatic Biodiversity (BIOAQUA) of the IZSPLV (Avigliana, Turin, Italy), where they were acclimated for two weeks.

To mitigate aggressive behavior, individuals were housed separately in 2 L tanks, each equipped with an independent recirculation pump. Artificial seawater was prepared using commercial salts (Haquoss Hi-Tech Reef Salt; Haquoss, Turin, Italy) at a concentration of 20 g/L. The solution was allowed to equilibrate for 24 h prior to use to ensure standardization. The organisms were maintained under a 16 h light and 8 h darkness photoperiod and provided with a commercial diet (Optiline K2P; Skretting, Verona, Italy) twice weekly. To prevent the accumulation of ammonia and other metabolic by-products, a 100% water exchange was performed twice per week, approximately 2 h after feeding. This maintenance protocol was established through preliminary trials, which confirmed that full renewals twice weekly under these conditions ensured stable physicochemical parameters and minimized waste accumulation throughout the experimental period.

### 2.2. Experimental Design and Exposure to PVA

Currently, the scientific literature lacks comprehensive data on environmental concentrations of PVA [23]. Nevertheless, PVA has been detected in various environmental matrices and is recognized as one of the most prevalent water-soluble polymers (WSPs) in aquatic ecosystems. Based on the study by Suaria et al. [14], and considering its relative abundance (1.2%), PVA corresponds to approximately 0.015 ± 0.019 particles m^−2^. This is particularly relevant in the Mediterranean Sea, especially in accumulation areas such as the Adriatic basin [14]. However, this approach may underestimate its actual environmental presence, as dissolved or colloidal fractions of PVA would not be captured by particle-based sampling and should more appropriately be expressed in mass-based units (e.g., µg/L). Given the lack of environmental relevant concentrations and the need to investigate potential toxicological mechanisms, the concentrations applied in the present study were intentionally selected to be higher than those reported in the environment. This approach allowed for the exploration of sublethal mechanistic effects of PVA in the selected model organism under controlled conditions. Furthermore, the tested concentrations were chosen to be comparable with those used in recent experimental studies on other aquatic organisms, i.e., refs. [21,22].

The PVA for the experiment was purchased by Sigma-Aldrich, Prague, Czech Republic (CAS number: 9002-89-5). The compound appeared as white or off-white crystals with a molecular weight of the particles between 8.000 and 10.000 Da and 80% hydrolysis. A stock solution containing salt water at 20 psu and PVA at a concentration of 5.3 g L^−1^ was prepared for use in the experiment. From this stock, exposure treatments of 0.5 mg L^−1^ (C1), 5 mg L^−1^ (C2), and 25 mg L^−1^ (C3) were prepared, along with a control group maintained in plain salt water. The selection of this specific PVA was made with the objective of conducting a comparative analysis with previous studies that utilized the same material [21,22].

**Figure 2:**
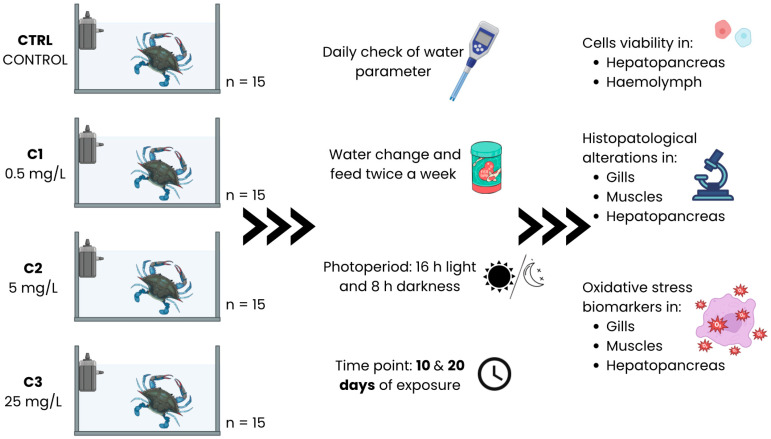
Schematic representation of the experimental design.

After the acclimation period, 15 specimens of *C. sapidus* were exposed to each experimental concentration, with each individual housed separately in 20 × 13 × 11 cm containers (2 L capacity). Individuals were randomly assigned to the different experimental treatments to minimize potential biases related to biological variability. Feeding regimes and water conditions were kept consistent with those used during the acclimation period. Daily, water parameters were checked with a multiparameter probe (Hanna Instruments^®^, Woonsocket, RI, USA; model HI98494); particularly, temperature (°C), dissolved oxygen (mg L^−1^), salinity, and pH (units) were recorded (Figure 2). Ammonia and nitrite levels in the water were monitored daily using a multiparameter photometer (HI83300; Hanna Instruments, USA) following the manufacturer’s instructions.

Furthermore, complete water renewal was carried out twice a week to maintain a consistent PVA concentration in the solution, as a low degradation rate was expected due to the absence of pretreatment and the lack of additives or co-formulants that could enhance its solubility or biodegradability [23].

Crabs were sampled after 10 and 20 days of exposure. Hemolymph was collected using sterile 1 mL syringes, while tissues were excised with sterile scissors and scalpels for oxidative stress biomarker determination and histological analysis. Animals were euthanized by immersion in ice water, following standard procedures for decapod crustaceans [48,49].

### 2.3. Hemocytes and Hepatocytes Cell Viability

To evaluate cell viability and membrane integrity, cytotoxicity assays were performed on both hemolymph and hepatopancreas cells. Hemolymph (at least 300 μL per crab) was collected from the unsclerotized membrane between the abdomen and carapace or from the leg joints using a 1 mL plastic syringe. Samples were transferred to Eppendorf tubes and diluted at a 1:1 ratio with an anticoagulant solution containing citrate buffer and EDTA (0.45 M NaCl, 0.1 M glucose, 30 mM sodium citrate, 26 mM citric acid, and 10 mM EDTA, pH 4.6), then stored at 4 °C [50].

Lysosomal membrane stability was determined through the Neutral Red retention assay, while cell membrane integrity was assessed using the Trypan Blue exclusion test [51,52,53].

A Neutral Red stock solution (4 mg/mL) was prepared in DMSO and stored at 4 °C in the dark until use. For the hemolymph analyses, due to the use of the anticoagulant solution, slight modifications were made to Impellitteri et al.’s [53] methodology to adapt the methods to the pH sensibility of the Neutral Red stain. The stock solution was diluted 1:100 in calcium and magnesium-free isomotic medium (CMFS) at 700 mOsm (NaCl 330 mM; KCl 10 mM; Hepes 20 mM) to obtain a working solution at 40 µg/mL Neutral Red and 1% DMSO, consistent with concentrations commonly applied in crustacean hemocyte assays [50,54,55,56]. Neutral Red is a weak cationic dye whose uptake is favored under near-natural extracellular conditions and markedly reduced in acidic media [57]. The working solution was added to hemocytes at a 3:1 ratio (*v*/*v*) to ensure suitable conditions for dye permeation and consistent staining. Cells were incubated for 15 min. at room temperature in the dark and examined immediately under light microscopy. Hemocytes showing distinct lysosomal accumulation of the dye were classified as viable, while diffuse cytoplasmic staining indicated lysosomal destabilization.

For the Trypan Blue exclusion test, cell membrane integrity was assessed using a standard vital dye approach. Non-viable cells, characterized by compromised plasma membranes, appeared blue-stained, while viable cells excluded the dye [58].

The hepatopancreas was carefully dissected, finely minced on a glass slide in CMFS and enzymatically dissociated with 0.01% collagenase type IV (175 U mg^−1^, Sigma-Aldrich, Milan, Italy) for 60 min at 18 °C in a thermostatic water bath. The resulting cell suspension was sequentially filtered through 200 μm and 50 μm nylon meshes to remove undigested debris, and resuspended in a physiological isotonic solution (700 mOsm) according to Multisanti et al. [22] with slight modifications to adapt the solution to the physiological status of *C. sapidus* (NaCl 265 mM; KCl 10 mM; CaCl_2_ 5 mM; MgCl_2_ 35 mM; Hepes 20 mM; Glucose 10 mM) in accordance with Holt and Kinsey [59]. The filtered solution was centrifuged twice at 400× *g* for 10 min at 4 °C (Remi Elektrotechnik Ltd., NEYA 16R, Mumbai, India) and incubated for 1 h at 18 °C to allow for cell recovery before viability assessment.

Cell viability was expressed as the percentage of live cells (unstained by Trypan Blue or capable of retaining Neutral Red) relative to the total cell count, according to the following equation:

Cell viability (%) = (number of viable cells)/(total number of cells) × 100

### 2.4. Biomarkers of Oxidative Stress

Biomarker analyses were conducted on tissue samples (gills, hepatopancreas, and muscles) from each experimental concentration.

Protein extraction followed Magara et al. [60] using a buffer containing 50 mM phosphate and 2 mM EDTA (1:4 *w*/*v* buffer-to-sample ratio). Samples were homogenized with an Ultraturrax and centrifuged at 12,000× *g* for 12 min at 4 °C (Eppendorf AG, Centrifuge 5910 R, Wesseling, Germany). The supernatant was collected, aliquoted (2 mL), and stored in liquid nitrogen.

Oxidative stress biomarkers were measured in triplicate using a spectrophotometer (Peak Instruments, C-7100 UV/VIS, Düren, Germany) at 25 °C. Protein concentration was determined using the Lowry assay [61], which relies on the interaction between proteins and copper ions in alkaline conditions, followed by the reduction of the Folin–Ciocalteu reagent to form a colored complex. The absorbance of this complex, measured at 750 nm, is directly proportional to the protein content of the sample. The results were expressed as µg mL^−1^.

Superoxide dismutase (SOD) activity was determined according to Gao et al. [62], based on inhibition of pyrogallol autoxidation at 420 nm in the presence of EDTA (pH 8.2). One enzyme unit (U mL^−1^) corresponded to 50% inhibition of autoxidation. Activity was expressed as U mg^−1^ of protein.

Glutathione S-transferase (GST) activity was measured as described by Habig et al. [63], using 1-chloro-2,4-dinitrobenzene (CDNB, 60 µM) and glutathione (10 µM) in 0.1 M phosphate buffer (pH 6.5). Absorbance at 340 nm was recorded every 5 min, and activity calculated using CDNB’s extinction coefficient (9.6 mM^−1^ cm^−1^). Data were normalized to protein content and expressed as nmol (µg·min)^−1^.

Glutathione peroxidase (GPx) activity followed the work of Badary et al. [64], using protein extracts incubated with 10 mM GSH, 2.4 U mL^−1^ glutathione reductase, and 1.5 mM NADPH. After 5 min at 37 °C in the presence of H_2_O_2_, absorbance at 340 nm was recorded for 2 min. Activity was expressed as nmol (mg·min)^−1^, normalized to protein content.

Lipid peroxidation was assessed through malondialdehyde (MDA) quantification following Uchiyama and Mihara [65]. Protein extracts (10%) were reacted with 1% phosphoric acid and 0.6% thiobarbituric acid, heated at 100 °C, cooled, and extracted with butanol. The MDA–TBA complex was read at 535 nm, and the results were expressed as µmol mg^−1^ protein.

### 2.5. Histological Analyses

Tissue samples (muscle, gills, and hepatopancreas) were fixed in 10% neutral buffered formalin, dehydrated in a graded ethanol series, and embedded in paraffin. Sections of 4+/−2 µm thickness were obtained using a microtome and mounted on glass slides. Sections were stained with hematoxylin and eosin (H&E) following standard histological procedures. Histological observations were performed using a light microscope (Nikon Eclipse 80i, Nikon Instruments Inc., Amstelveen, The Netherlands) equipped with a digital camera. Images were acquired using Nis Elements software (version 6.10.01) and processed only for brightness and contrast adjustment without altering the original content.

### 2.6. Statistical Analysis

Data were first examined for normality and homogeneity of variance using the Shapiro–Wilk and Levene’s tests. Because distributions deviated from normality or sample sizes were small, non-parametric methods were used. Differences in the percentage of viable cells in hemolymph and hepatopancreas among exposure concentrations and days were assessed using the Kruskal–Wallis test, followed by Dunn’s post hoc test [66] for pairwise comparisons.

Potential variations in oxidative stress biomarkers (CTRL, C1, C2, and C3) were similarly evaluated using the Kruskal–Wallis test, with significant results further explored through Dunn’s post hoc comparisons.

The integrated biochemical response to PVA exposure was analyzed using the Threshold-based Integrative Biomarker Response (IBR-T) index, calculated in R via the dplyr and tidyr packages [67]. For each tissue, mean values of selected biomarkers (SOD, GPx, GST, and MDA) were calculated per treatment. Reference and threshold values were established from the control group (CTRL) using the median and the 95th percentile, respectively. For each treatment, the log-ratio between the biomarker mean and its reference value was computed and standardized using the standard deviation across treatments. Only biomarkers exceeding the threshold were included in the final IBR-T calculation. The IBR-T score for each treatment represented the mean of the absolute standardized log-ratio values of significantly induced biomarkers. This method provided a quantitative and comparative evaluation of biomarker responses among treatments and tissues, ensuring both statistical robustness and biological relevance [67].

Non-metric multidimensional scaling (NMDS) was performed to visualize the multivariate patterns in the cellular biomarker dataset, including measures of vitality and antioxidant enzyme activities. Prior to NMDS, missing values were imputed using a multivariate imputation method using the R package missMDA [68]. Data were then standardized (z-score) to account for differences in scale across biomarkers and to stabilize variances. Euclidean distance was used as the dissimilarity metric for NMDS, rather than Bray–Curtis, due to the high prevalence of zero and near-zero values present in the dataset. NMDS was conducted with two dimensions (k = 2) and a maximum of 100 random starts to ensure convergence. Sample scores were extracted for visualization, and categorical variables (Matrix, Day, Treatment) were overlaid in the final plots using the ggplot2 package. The stress value was used to assess the goodness of fit, with values below 0.2 considered acceptable [69].

For the application of the factorial linear model, biomarker data were first standardized using z-score transformation to account for differences in measurement scales, improve comparability among variables, and stabilize variances. Subsequently, a factorial linear model was fitted including treatment (CTRL, C1, C2, C3), exposure time (10 and 20 days), biological matrix (hemolymph, hepatopancreas, muscle, and gills), and biomarker type as fixed factors. Interactions among treatment, exposure time, and biomarker type were also included to account for potential biomarker-specific temporal responses. The robustness of the model was confirmed through visual inspection of residual plots and homogeneity of variance tests; the design was considered resilient to moderate departures from normality due to its balanced nature and sample size. Finally, the significance of the factors was determined via Type III ANOVA using the “car” package in R. All statistical analyses were performed in R Studio (version 2023.12.1 Build 402) [70,71]. Statistical significance was set at *p* < 0.05.

## 3. Results

### 3.1. Water Physicochemical Parameters

The physicochemical parameters of the water were monitored daily throughout the exposure of *Callinectes sapidus* to PVA and are summarized in Table 1.

Water temperature ranged from 23.59 ± 2.34 °C in the CTRL group to 24.18 ± 2.46 °C in C1. The pH values varied between 8.01 ± 0.32 in C2 and 8.03 ± 0.16 in C1. Dissolved oxygen concentrations ranged from 5.74 ± 1.36 ppm in C3 to 6.35 ± 0.68 ppm in C1. Salinity, expressed in psu, ranged between 16.93 ± 0.51 in C1 and 17.08 ± 0.61 in CTRL.

Throughout the experiment, ammonia concentrations remained below the detection limit, while nitrite (NO_2_^−^) levels ranged from 0 to 0.01 mg L^−1^.

### 3.2. Mortality and Biometric Features

No visible behavioral alterations were observed in *C. sapidus* across any of the treatments, including the highest tested concentration. All individuals remained active, responsive to stimuli, and showed no apparent signs of distress or mortality. Moreover, no molting events were observed during the exposure period. No significant differences were observed in biometric features (*p* > 0.05) among the experimental groups (C1, C2, C3) and the control (CTRL), as shown in Table 2 and in Figure S1.

Although sex was not used as a factor in the experimental design, its distribution across treatments was recorded and is reported in Figure S2. Moreover, none of the females utilized in the experiment were observed to be ovigerous.

### 3.3. Effects on Hemolymph and Hepatopancreas Cell Viability

Hemolymph (HE) and hepatopancreas (HP) cell viability of *C. sapidus* after exposure to different PVA concentrations for 10 and 20 days is reported in Table S1.

**Figure 3:**
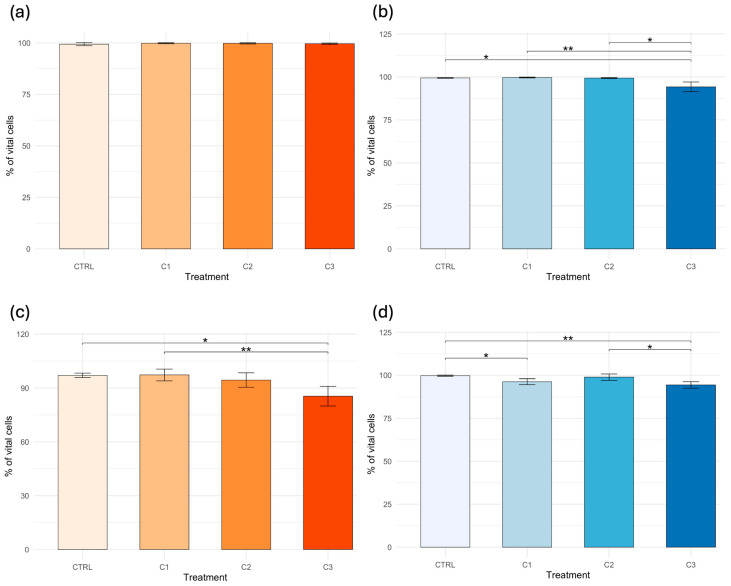
Cell viability (%) of *Callinectes sapidus* after 10 days of exposure to PVA. Panels show: (**a**) Neutral Red (NR) assay in hemolymph; (**b**) Trypan Blue (TB) assay in hemolymph; (**c**) NR assay in hepatopancreas; and (**d**) TB assay in hepatopancreas. Asterisks indicate significant differences among treatments according to the Kruskal–Wallis test and Dunn’s post hoc test (* *p* < 0.05; ** *p* < 0.01).

Cell viability remained high across treatments, particularly in hemolymph samples, where values consistently exceeded 99% in both assays (TB and NR). In contrast, hepatopancreas samples showed greater variability. After 10 days, a slight decrease in HP cell viability was observed at the highest concentration (C3), particularly in the NR assay (85.46 ± 5.84%), while values remained close to 95–99% in the other groups. No significant differences were detected among treatments in HE using the NR assay (*p* > 0.05). Conversely, the Kruskal–Wallis test revealed significant effects among treatments in the TB assay in HE (*p* = 0.0018), as well as in both NR (*p* = 0.0042) and TB (*p* = 0.0010) assays in the HP. Pairwise comparisons performed with Dunn’s post hoc test are illustrated in Figure 3, where different numbers of asterisks indicate increasing levels of statistical significance among groups.

**Figure 4:**
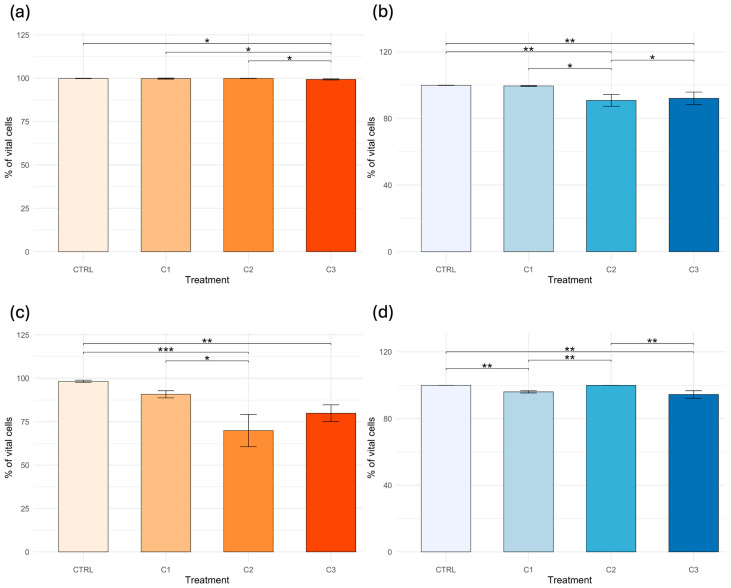
Cell viability (%) of *Callinectes sapidus* after 20 days of exposure to PVA. Panels show: (**a**) Neutral Red assay (NR) in hemolymph; (**b**) Trypan Blue (TB) assay in hemolymph; (**c**) NR assay in hepatopancreas; and (**d**) TB assay in hepatopancreas. Asterisks indicate significant differences among treatments according to the Kruskal–Wallis test and Dunn’s post hoc test (* *p* < 0.05; ** *p* < 0.01; *** *p* < 0.001).

After 20 days, the NR method indicated a marked reduction in HP cell viability at C2 (69.90 ± 9.30%) and C3 (79.90 ± 4.83%), whereas the TB assay showed only minor decreases. The Kruskal–Wallis test revealed significant effects among treatments in the NR and TB assay in the HE (respectively, *p* = 0.01247 and *p* = 0.0002398), as well as in both the NR (*p* = 0.000129) and TB (*p* = 0.000152) assays in the HP. Pairwise comparisons performed with Dunn’s post hoc test are illustrated in Figure 4, where different numbers of asterisks indicate increasing levels of statistical significance among groups.

A selection of explanatory images showing cells subjected to the Trypan Blue (TB) exclusion method and the Neutral Red (NR) retention assay are provided in the Supplementary Materials (Figure S3).

### 3.4. Histological Observations

Microscopic analyses were performed to evaluate pathological changes in three tissues (muscle, gills, and hepatopancreas) collected after 10 and 20 days of exposure. All tissues were examined microscopically at different magnifications. No histopathological lesions were observed in the muscle tissue of exposed animals at either sampling time.

Muscle fibers were arranged in compact and well-organized bundles, separated by thin connective tissue layers. Individual fibers appeared elongated and cylindrical, with clearly visible cross striations corresponding to the regular arrangement of actin and myosin filaments. The sarcoplasm was homogeneous and moderately eosinophilic, while nuclei were generally located at the periphery of the fibers and displayed regular morphology, with no evidence of pyknosis or degeneration.

In all analyzed samples, muscle fibers appeared well organized, maintaining their normal morphology and structural integrity. No evidence of degeneration, necrosis, inflammatory infiltration, or structural disorganization was detected. These observations suggest that muscle tissue was not significantly affected by PVA exposure under the experimental conditions.

**Figure 5:**
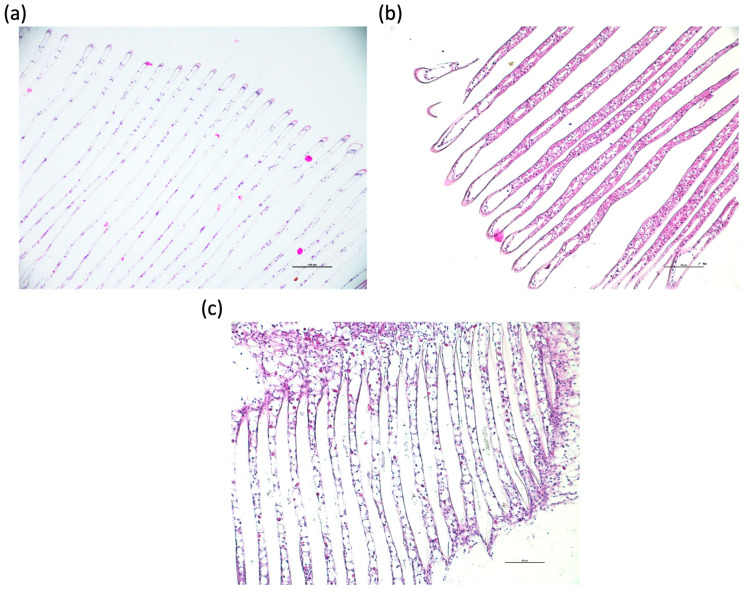
Gill lamellae of *Callinectes sapidus*: (**a**) control animals; (**b**) animals exposed to the highest concentration (C3: 25 mg L^−1^) after 10 days; (**c**) animals exposed to C3 after 20 days. Bar scale: (**a**) 100 µm; (**b**) and (**c**) 50 µm.

In contrast, gill tissues showed several alterations that appeared to be related to the duration of exposure. The gills of control crabs exhibited the typical crustacean branchial architecture, as shown in Figure 5a. Each lamella was lined by a thin epithelium composed mainly of flattened epithelial cells, which facilitates efficient gas exchange between the hemolymph and the surrounding water. The lamellar epithelium appeared continuous and regular.

In crabs exposed for 10 days, the gills displayed epithelial cell hyperplasia, resulting in a thickening of the respiratory epithelium and a consequent reduction in the available surface area for gas exchange (Figure 5b). This condition may represent a defensive response aimed at limiting the penetration of external stressors or contaminants through the gill epithelium.

After 20 days of exposure, the gills exhibited a different morphological pattern, suggesting a more chronic or adaptive response (Figure 5c). In these specimens, the epithelial cells appeared thinner and more similar to those observed in control animals, indicating a partial restoration of the epithelial architecture. However, a marked increase in eosinophilic cells was observed within the gill tissue. The presence and accumulation of these cells are generally associated with prolonged stress conditions and may reflect an ongoing detoxification process or a chronic physiological response to sustained exposure to PVAs.

The hepatopancreas of control animals showed the typical tubular organization. The hepatopancreatic tubules were lined by a single layer of epithelial cells, arranged around a central lumen. The epithelial cells appeared with normal cytoplasmic morphology and basally located nuclei. In exposed animals, the hepatopancreas did not show marked structural alterations compared to control specimens. The general architecture of the tubules remained preserved. Only mild cellular vacuolization was observed in some epithelial cells, which may reflect metabolic activity related to detoxification processes. Overall, these observations suggest that, under the present experimental conditions, the hepatopancreas maintained its structural integrity and did not exhibit severe histopathological damage following exposure to PVAs.

### 3.5. Oxidative Stress Biomarkers

#### 3.5.1. Oxidative Stress in Gills

**Figure 6:**
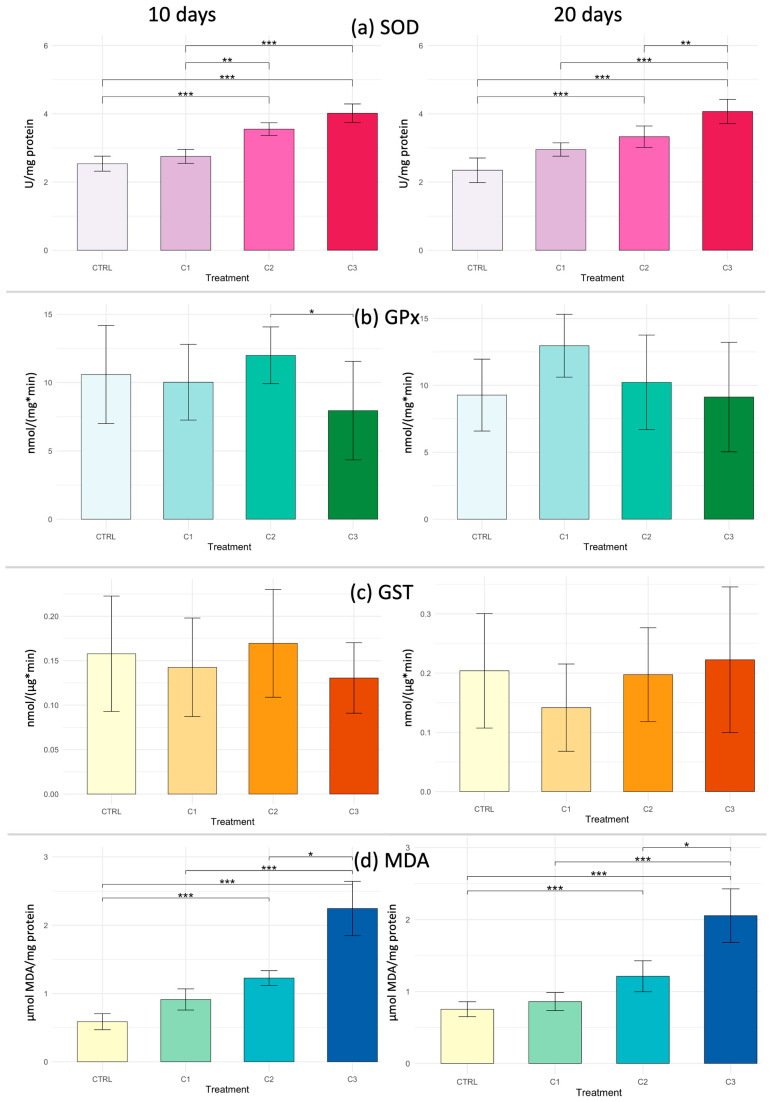
Biomarker responses in gills of *Callinectes sapidus* exposed to three concentrations of PVA (C1: 0.5 mg L^−1^; C2: 5 mg L^−1^; C3: 25 mg L^−1^) compared to control (CTRL) after 10 and 20 days. Biomarker analyzed: (**a**) SOD = superoxide dismutase; (**b**) GPx = glutathione peroxidase; (**c**) GST = glutathione S-transferase and; (**d**) MDA = malondialdehyde. Asterisks indicate significant differences among treatments according to the Kruskal–Wallis test and Dunn’s post hoc test (* *p* < 0.05; ** *p* < 0.01; *** *p* < 0.001).

After 10 days, in the gills of *Callinectes sapidus*, the Kruskal–Wallis test revealed significant differences among treatments for SOD (*p* = 1.23 × 10^−8^), GPx (*p* = 0.0261), and MDA (*p* = 4.50 × 10^−9^). Pairwise comparisons conducted using Dunn’s post hoc test are shown in Figure 6, where different numbers of asterisks denote increasing levels of statistical significance among groups. Only GST activity showed no statistically significant variation (*p* > 0.05). SOD activity increased progressively with PVA concentration, showing highly significant differences between CTRL and both C2 and C3 groups. GPx activity displayed moderate variability, with significantly higher values in C2 compared to C3. MDA levels, used as a marker of lipid peroxidation, exhibited a clear dose-dependent increase, with C3 showing the highest values and statistically significant differences compared to all other treatments.

After 20 days of exposure to PVA, the Kruskal–Wallis test revealed statistically significant differences in SOD activity (*p* = 2.26 × 10^−8^) and MDA content (*p* = 7.46 × 10^−9^). SOD activity increased markedly with PVA concentration, with significantly higher values in C2 and C3 compared to CTRL and C1. MDA levels exhibited a strong concentration-dependent increase, with the highest values recorded in C3, significantly different from all other treatments. GPx and GST activities showed no statistically significant variation (*p* > 0.05).

#### 3.5.2. Oxidative Stress in Hepatopancreas

**Figure 7:**
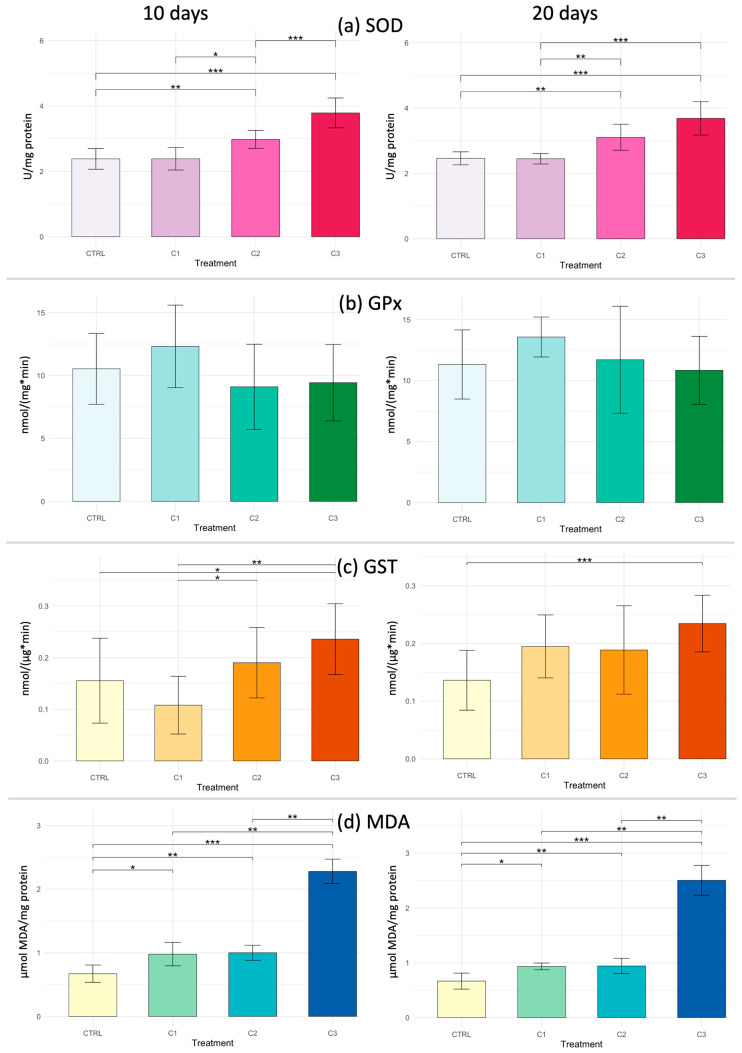
Biomarker responses in hepatopancreas of *Callinectes sapidus* exposed to three concentrations of PVA (C1: 0.5 mg L^−1^; C2: 5 mg L^−1^; C3: 25 mg L^−1^) compared to control (CTRL) after 10 and 20 days. Biomarker analyzed: (**a**) SOD = superoxide dismutase; (**b**) GPx = glutathione peroxidase; (**c**) GST = glutathione S-transferase and; (**d**) MDA = malondialdehyde. Asterisks indicate significant differences among treatments according to the Kruskal–Wallis test and Dunn’s post hoc test (* *p* < 0.05; ** *p* < 0.01; *** *p* < 0.001).

In the hepatopancreas, the Kruskal–Wallis test revealed significant differences among treatments after 10 days of exposure for SOD (*p* = 1.51 × 10^−7^), GST (*p* = 0.0038), and MDA (*p* = 6.16 × 10^−8^). Pairwise comparisons performed using Dunn’s post hoc test are presented in Figure 7. SOD activity showed a concentration-dependent increase, with significantly higher values in C2 and C3 compared to CTRL and C1. GST activity also increased progressively with exposure, reaching significantly higher levels in C2 and C3 compared to CTRL. MDA content displayed the most pronounced response, with a strong and significant elevation in C3 relative to all other treatments, as shown in Figure 7. Finally, only GPx activity showed no statistically significant variation (*p* > 0.05).

After 20 days of exposure, the Kruskal–Wallis test revealed statistically significant differences in SOD (*p* = 4.74 × 10^−7^), GST (*p* = 0.0012), and MDA (*p* = 4.68 × 10^−8^). Dunn’s post hoc test indicated that SOD and MDA levels increased significantly, particularly in the highest concentration group (C3), suggesting enhanced oxidative stress at prolonged exposure.

#### 3.5.3. Oxidative Stress in Muscle

**Figure 8:**
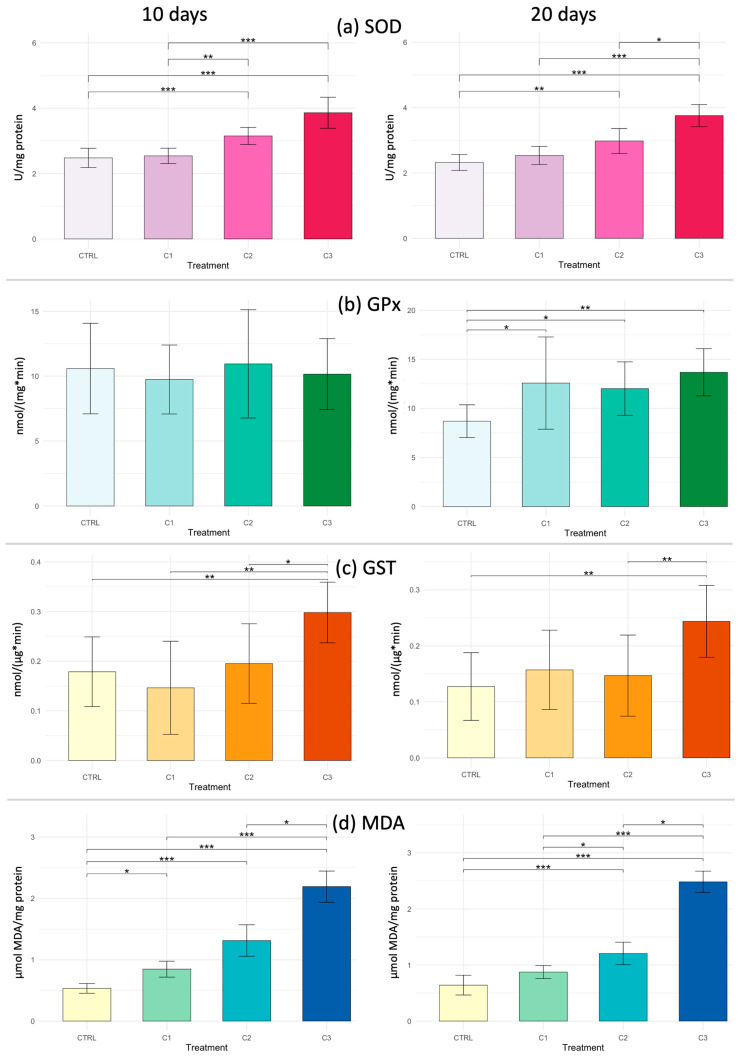
Biomarker responses in muscle of *Callinectes sapidus* exposed to three concentrations of PVA (C1: 0.5 mg L^−1^; C2: 5 mg L^−1^; C3: 25 mg L^−1^) compared to control (CTRL) after 10 and 20 days. Biomarker analyzed: (**a**) SOD = superoxide dismutase; (**b**) GPx = glutathione peroxidase; (**c**) GST = glutathione S-transferase and; (**d**) MDA = malondialdehyde. Asterisks indicate significant differences among treatments according to the Kruskal–Wallis test and Dunn’s post hoc test (* *p* < 0.05; ** *p* < 0.01; *** *p* < 0.001).

The Kruskal–Wallis test revealed statistically significant differences between the treatments for SOD, GST and MDA (*p* = 4.108 × 10^−8^, *p* = 0.00122 and *p* = 2.134 × 10^−9^, respectively). Pairwise comparisons performed using Dunn’s post hoc test are presented in Figure 8. Overall, SOD and GST activity increased progressively with exposure, reaching significantly higher levels in C2 and C3 compared to CTRL. Concurrently, MDA levels exhibited an increase, following a concentration-dependent pattern. Only GPx activity exhibited a lack of statistically significant variation (*p* > 0.05).

Finally, the muscle tissue after 20 days of exposure showed a statistically significant difference in all biomarkers analyzed: SOD (*p* = 1.47 × 10^−7^), GPx (*p* = 0.00141), GST (*p* = 0.001974) and MDA (*p* = 5.28 × 10^−9^). A clear dose-dependent pattern can be observed, particularly for SOD and MDA activities, which increased significantly at higher exposure levels (C2 and C3). GPx and GST also showed significant elevations compared to the control, indicating activation of the antioxidant defense system. The rise in MDA levels at the highest concentrations suggests enhanced lipid peroxidation, confirming oxidative stress induced by PVA exposure, as shown in Figure 8.

### 3.6. IBR-T Index

**Figure 9:**
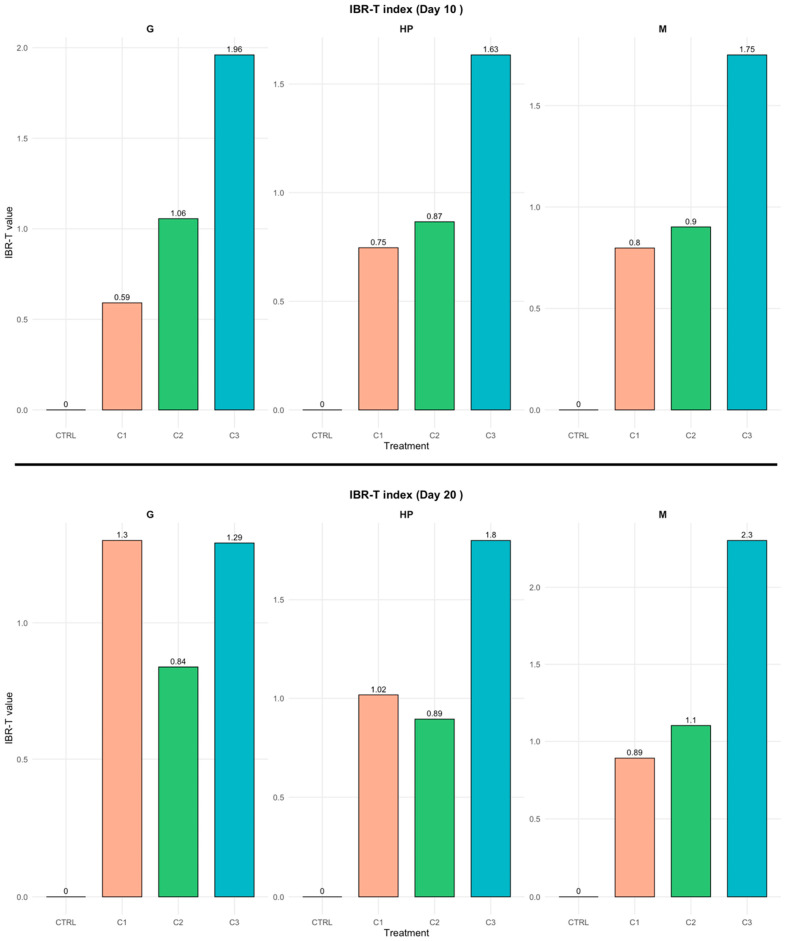
Integrated Biomarker Response—Threshold (IBR-T) index values in gills (G), hepatopancreas (HP), and muscle (M) of *Callinectes sapidus* after 10 and 20 days of exposure to increasing PVA concentrations.

The IBR-T index, representing the biochemical response profiles of *C. sapidus*, revealed differential biochemical responses among the three analyzed tissues across the various treatments and times, as summarized in Figure 9 and Tables S2 and S3.

After 10 days, the IBR-T index increased progressively with concentration in all tissues, reaching the highest values in C3 (G: 1.96; HP: 1.63; M: 1.75). This pattern indicates an early activation of the antioxidant and detoxification responses under higher exposure levels. After 20 days, the IBR-T values remained elevated, particularly in the muscle (2.3) and hepatopancreas (1.8), suggesting cumulative stress effects over time. Overall, the IBR-T index highlighted a concentration- and time-dependent response to PVA exposure, with the strongest biological alterations occurring in the highest treatment (C3).

### 3.7. NMDS Analysis

**Figure 10:**
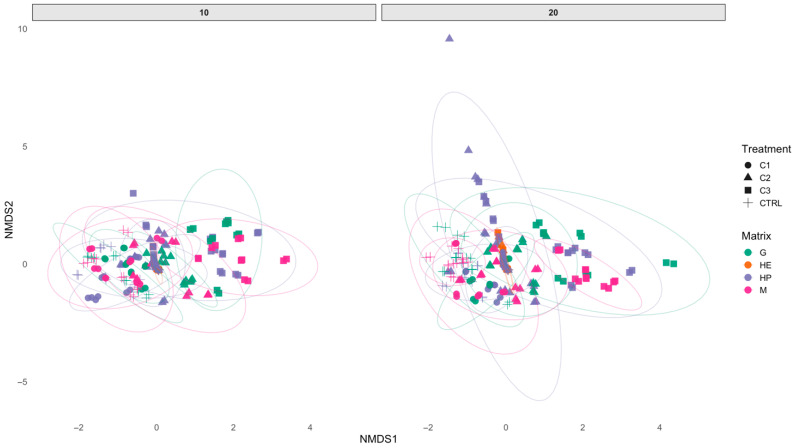
Non-metric multidimensional scaling (NMDS) plot of total biomarkers and cell viability in *Callinectes sapidus* different tissues. Biomarkers and cell viability are clustered according to treatment concentrations.

The NMDS analysis (stress = 0.1488) indicates a sufficiently robust data structure, as shown in Figure 10.

At 10 days, no clear separation among treatments is observed, with substantial overlap of the ellipses and limited clustering. At 20 days, however, a greater dispersion becomes evident, particularly in samples exposed to C2 and C3, suggesting increased biological variability associated with chemical stress. Tissue matrices contribute significantly to the separation within the NMDS space, with gills and hepatopancreas showing the highest variability, whereas muscle samples tend to cluster more centrally and remain less dispersed. Hemolymph vitality appears more overlapped with other compartments, indicating a lower discriminative power.

### 3.8. Factorial Linear Model

The model revealed a significant overall effect of PVA exposure on the physiological status of *C. sapidus* (Treatment: F_3_,1301 = 3.06, *p* = 0.027). Biomarker type significantly influenced the observed responses (F_4_,1301 = 13.28, *p* < 0.001), indicating substantial variability among the measured biomarkers. Similarly, the biological matrix had a strong effect on biomarker responses (F_3_,1301 = 14.45, *p* < 0.001), highlighting tissue-specific physiological differences among hemolymph, hepatopancreas, muscle, and gills.

Exposure time alone did not produce a significant overall effect (Day: F_1_,1301 = 1.99, *p* = 0.158).

However, a significant interaction between Treatment and Day was detected (F_3_,1301 = 5.81, *p* = 0.0006), indicating that the effect of PVA exposure varied across sampling times. The Treatment × Biomarker interaction was highly significant (F_12_,1301 = 25.08, *p* < 0.001), demonstrating that different biomarkers responded differently to increasing PVA concentrations. Furthermore, a significant Treatment × Day × Biomarker interaction (F_12_,1301 = 2.32, *p* = 0.006) indicated that biomarker responses to PVA exposure varied over time, showing clear dose-dependent trends, with temporal variations depending on biomarker type and exposure conditions.

Overall, these results reveal a complex physiological response characterized by tissue-specific variability and biomarker-dependent responses to PVA exposure.

## 4. Discussion

### 4.1. Experimental Conditions and Water Parameters

The physicochemical water parameters used in this study reflected the environmental conditions of the Sacca di Goro during the sampling sessions in June 2025, but also of previous years [47]. Temperature and salinity are key factors influencing the health and well-being of these animals. The experimental conditions in which the crabs were maintained closely matched the optimal ranges identified in recent studies for this species [72,73]. Maintaining these consistent conditions provided a controlled experimental environment, ensuring that the physiological and biochemical responses observed could be confidently attributed to PVA exposure rather than to natural variability among individuals or to other environmental stressors [74]. Additionally, the renovation of water with PVA ensured the maintenance of constant concentrations throughout the duration of the experiment. This condition exhibited similarity to PVA in a marine environment, attributable to its scarce degradability, as demonstrated by various studies [23,75].

### 4.2. Hemolymph and Hepatopancreas Cell Viability

In the *C. sapidus*, hemocytes represent a key component of the immune systems of these crustaceans; the cellular composition was comparable to that observed in other crab species, such as the Mediterranean green crab *Carcinus aestuarii* [50,76]. The main cells observed, indeed, were hyalinocytes, semigranulocytes and granulocytes that have a rounded or oval shape [77]. Changes in hemocyte number may indicate stress or contaminant exposure. In this regard, the results obtained, both after 10 and 20 days of exposure, are in line with the literature about PVA toxicity and the *C. sapidus* hemolymph characteristics. In particular, the NR assay, after 10 days of exposure, confirms high cell viability, as previously highlighted by Fabrello et al. [77]. This is consistent with high cell viability and absence of membrane or lysosomal instability. Nevertheless, the reduction in viability observed after the 20-day exposure suggests a time- and dose-dependent effect of the PVA presence in the water, as also shown by the few studies conducted on the same contaminants and invertebrate models, such as the *Mytilus galloprovincialis* and the *Paracentrotus lividus*, in which low viability and higher mortality were reported at the highest concentration tested and the prolonged exposure time [22,23]. Similar results are those observed with the Trypan Blue assay, in which lower viability was reported in the highest concentration tested, reinforcing the idea of a dose- and time-dependent effect of PVA on *C. sapidus* cells.

Lysosomal instability and reduced cell viability were more evident in hepatopancreas analyses, in which 20-day exposure showed a decline in viability compared to the control group. The hepatopancreas is considered the main metabolic center of decapod crustaceans and plays a central role in energy storage, digestive enzyme production, immune defense, and xenobiotic processing [78]. The elevated cellular depth recorded in the C2 and C3 groups in NR and TB assays underscores a negative interaction between the model organisms and the PVA compounds. Furthermore, the results obtained align with those previously observed in other invertebrate model species tested with PVA concentrations comparable to those selected for the present study [22]. Moreover, analogous findings of cytotoxicity were reported in other water-soluble polymer studies where prolonged exposure compromises the cellular homeostasis capability [79,80]. However, the lack of information present in the literature, to our knowledge, on the topic makes further analysis necessary for a better understanding of the defense mechanisms implemented by *C. sapidus* after physiological damage.

### 4.3. Histopathological Lesions

The histopathological alterations observed in the examined tissues suggest a time-dependent adaptive response to the PVA exposure. Muscle tissue showed no detectable changes, possibly due to its lower metabolic turnover relative to organs directly exposed to environmental interactions, whereas the gills underwent evident morphological remodeling.

In contrast, the gills exhibited clear morphological modifications associated with the duration of exposure. The gill epithelium represents the primary interface with the environment and is among the first tissues to respond to waterborne stressors and contaminants. Besides respiration, gills are also involved in ion regulation and in the initial uptake or exclusion of xenobiotics, making them particularly sensitive indicators of environmental stress [81].

After 10 days of exposure, epithelial hyperplasia was observed, resulting in thickening of the respiratory epithelium and a reduction in the lamellar surface available for gas exchange. This epithelial proliferation is widely described as a protective response in aquatic organisms exposed to pollutants. The increased cellular thickness may function as a barrier that limits the penetration of potentially harmful substances across the branchial epithelium, although it may simultaneously reduce respiratory efficiency. Similar hyperplastic responses in gill tissues have been reported in crustaceans exposed to various environmental stressors, including ammonia and heavy metals [82]. After 20 days of exposure, the gill morphology showed a partially different pattern. The respiratory epithelium appeared thinner and more comparable to that observed in control specimens, suggesting a structural reorganization possibly aimed at restoring respiratory efficiency. However, this apparent normalization was accompanied by a marked increase in the number of cells in the gill tissue. The accumulation of these cells is possibly associated with immune activation and prolonged stress responses in aquatic invertebrates and may reflect an ongoing physiological response to sustained exposure to the stressor. Their presence may therefore indicate the activation of cellular defense mechanisms involved in immune regulation and detoxification processes.

In the present study, the general tubular architecture of the hepatopancreas remained preserved, although moderate cellular vacuolization and slight variations in cytoplasmic appearance were occasionally observed in exposed specimens. These alterations may reflect physiological adjustments associated with metabolic or detoxification activity rather than severe pathological damage. Previous studies have shown that crustacean hepatopancreatic cells can undergo reversible structural modifications in response to environmental stressors as part of adaptive detoxification mechanisms. This apparent discrepancy between biochemical and histological responses likely reflects the different levels of biological organization involved, with oxidative stress biomarkers acting as early and sensitive indicators of physiological imbalance, while tissue level alterations generally require longer exposure to develop [83]. The relatively mild and partially reversible histological changes observed here may therefore be explained by the activation of effective antioxidant and detoxification mechanisms, which can temporarily mitigate cellular damage and delay the onset of more severe structural impairment.

Overall, the histological findings suggest that *C. sapidus* responds to PVA exposure mainly through adaptive physiological mechanisms rather than through severe tissue damage. The absence of alterations in muscle tissue, together with the structural remodeling observed in gills and the mild responses detected in the hepatopancreas, supports the hypothesis that organs directly involved in environmental interaction and metabolic regulation play a key role in mediating the organism’s response to PVA exposure. A limitation of the present study is the lack of quantitative morphometric analysis of histological alterations (e.g., epithelial thickness or cell density), which could provide a more robust evaluation of tissue responses. Future studies should integrate quantitative approaches to strengthen the interpretation of histopathological findings.

### 4.4. Interpretation of Biomarker Alterations

In response to environmental disturbances, cells may experience oxidative stress due to an imbalance between reactive oxygen species (ROS) production and endogenous antioxidant defense mechanisms. Elevated ROS can damage biological macromolecules, impairing cellular functions and potentially affecting organism health [84,85,86]. Oxidative stress biomarkers are widely used to assess contaminant effects in crustaceans, including metals and microplastics [82,87].

SOD acts as the first defense line against ROS by catalyzing the dismutation of superoxide anion into hydrogen peroxide (H_2_O_2_) [85]. In the present study, SOD activity was consistent across tissues and comparable to values reported for other crab species, such as *Carcinus aestuarii* [50]. GST and GPx responses were tissue- and time-dependent. GPx showed limited variation in most cases, except for an increase in muscle after prolonged exposure, suggesting a delayed antioxidant response. Despite the progressive activation of antioxidant enzymes, their activity may not have been sufficient to fully counteract oxidative processes under chronic high dose PVA exposure, suggesting a potential shift from adaptive responses toward adverse effects.

GPx responds to environmental factors and contaminant exposure [88,89,90]. By reducing peroxides via oxidation of GSH to GSSG, GPx prevents radical chain propagation and protects membranes [91,92]. In this study, GPx activity was unchanged in the hepatopancreas, reduced in gills at higher concentrations, and increased in muscle, suggesting tissue-specific modulation of H_2_O_2_ detoxification and lipid peroxidation [89].

GST catalyzes the conjugation of xenobiotics with GSH, supporting detoxification of electrophilic compounds and ROS scavenging [85,93,94,95,96]. Experimental studies conducted on *Chasmagnathus granulatus* have shown that this process also influences intracellular glutathione levels and redox balance [97]. Its efficiency depends on both GSH availability and ROS type, being more effective against peroxyl radicals (ROO•) than hydroxyl radicals (•OH) [98]. In this study, GST activity was limited in gills but active in hepatopancreas and muscle, consistent with tissue-specific metabolic roles. The hepatopancreas, as highly metabolic tissue, is characterized by high metabolic ROS production [78,82,99]. While it can initially limit lipid peroxidation, excessive ROS could overwhelm antioxidant defenses (SOD and GST), leading to membrane damage.

Unlike the gills and hepatopancreas, muscle tissue is not primarily involved in detoxification [49,100] but showed increased GST activity after 20 days at the highest PVA concentration, indicating a delayed activation of conjugation pathways, possibly linked to GPx-mediated H_2_O_2_ detoxification. Together, GPx and GST enzymes maintain redox homeostasis through complementary pathways linking antioxidant defense and biotransformation [101].

Potential interaction between PVA and the glutathione-dependent enzymes may be particularly relevant. PVA undergoes bioconjugation under physiological conditions, influenced by synthesis-related functional groups [102]. These properties suggest potential interactions with thiol-containing molecules such as GSH, potentially affecting the efficiency of GST and GPx-mediated detoxification processes and modulating biocompatibility. Further studies specifically targeting glutathione levels and related pathways would be needed to confirm this mechanism.

These interactions may contribute to alterations in ROS detoxification, as reflected by elevated SOD activity along with raised MDA levels in all PVA exposed crab tissues, indicating widespread oxidation of polyunsaturated fatty acids and potential physiological dysfunctions [103]. Similar oxidative responses have been reported in marine organisms exposed to different stressors [104,105], including mussels exposed to PVA [22], supporting the relevance of oxidative stress biomarkers for evaluating PVA induced effects. Overall, these results suggest a condition in which antioxidant defenses are activated in response to oxidative stress, but may become progressively less effective under higher exposure levels and longer durations, potentially leading to the onset of oxidative damage.

### 4.5. IBR-T Index and NMDS

The IBR-T index enabled the integration of multiple oxidative stress biomarkers into a single indicator of physiological alteration. Overall, IBR-T values increased with both PVA concentration and exposure time, indicating a dose- and time-dependent physiological response to PVA exposure in *C. sapidus*. After 10 days, the treated groups already exhibited moderate IBR-T values, particularly at the highest concentration, suggesting an activation of oxidative stress mechanisms. This response intensified after 20 days, with markedly higher IBR-T values across all tissues, especially in the hepatopancreas and gills. Distinct tissue-specific patterns were observed: the hepatopancreas showed the highest IBR-T values, consistent with its known physiological role, while gills also exhibited elevated values. Muscle generally displayed lower IBR-T values, although a pronounced increase was evident at the highest concentration after prolonged exposure. The increase in IBR-T was mainly driven by the combined contribution of antioxidant enzymes (SOD, GPx, GST) and lipid peroxidation, reflecting a potential imbalance between ROS production and detoxification capacity at higher PVA concentrations and longer exposure times. Notably, the simultaneous increase in both antioxidant activity and MDA levels suggests that although defense mechanisms were activated, they were not sufficient to fully counteract oxidative damage, indicating a shift from adaptive to potentially adverse physiological conditions.

The low NMDS stress value (0.1488) confirms a reliable two-dimensional representation of the dataset. NMDS ordination showed a clear separation between control and PVA-exposed samples, with treated groups progressively diverging from controls as concentration and exposure time increased. Controls clustered tightly, indicating stable physiological conditions, whereas higher-concentration treatments, particularly C2 and C3, exhibited greater dispersion, suggesting increased physiological variability and stress. Tissue-specific responses were evident, with hepatopancreas and gills showing greater separation from controls than muscle, confirming their higher sensitivity to oxidative stress. The increased dispersion observed at higher concentrations may further indicate heterogeneous individual responses, possibly reflecting differences in physiological resilience or detoxification capacity among organisms.

Despite relying on different analytical approaches, IBR-T and NMDS provided consistent and complementary results. IBR-T quantified the intensity of the stress response, while NMDS captured multivariate shifts in the overall biomarker profile. Treatments with higher IBR-T values corresponded to greater distances from the control cluster in the NMDS ordination, particularly at C3 and after 20 days. Together, these findings indicate that prolonged exposure to high PVA concentrations intensifies oxidative stress and induces coordinated multibiomarker responses, leading to a distinct physiological state clearly separated from controls. It should also be noted that the absence of earlier sampling points does not allow for the detection of potential acute responses occurring before 10 days, which may represent a more dynamic phase of physiological adjustment. The sustained increase in lipid peroxidation over time further suggests a persistent oxidative condition that could have downstream effects at higher levels of biological organization, potentially affecting organism fitness and, at a broader scale, population-level processes.

### 4.6. Factorial Linear Model Interpretation

The factorial linear model showed that exposure to PVA significantly affected the physiological status of *C. sapidus*, indicating that even relatively short time exposure can induce detectable sublethal effects. The treatment effect suggests that physiological alterations may still occur under experimental conditions and can be detected through biomarker-based approaches. These alterations should be interpreted with caution, as they may reflect early or compensatory responses rather than direct evidence of toxic effects, particularly in the absence of pronounced tissue damage or mortality.

The significant influence of biomarker type highlights the heterogeneous nature of the physiological responses, reflecting the activation of distinct physiological pathways. In addition, the strong effect of the biological matrix indicates clear tissue-specific responses, likely related to the distinct physiological roles of the analyzed organs. Sensitive tissues such as hepatopancreas and gills are expected to be more responsive to contaminant exposure [78,106].

Although exposure time alone did not significantly influence the overall response, the significant Treatment × Day interaction indicates that the physiological effects of PVA varied across sampling times, suggesting dynamic responses such as delayed stress effects or acclimation processes. Consistently, after 20 days of exposure, clear dose- and time-dependent trends were observed, with temporal variations depending on biomarker type and exposure conditions. Particularly, exposure to higher PVA concentrations resulted in a reduction in hepatopancreas cell viability, which could impair organ functionality under prolonged exposure. Considering the central role of the hepatopancreas in metabolic and detoxification processes in crustaceans, such alterations may have important implications for the long-term physiological condition of exposed organisms. However, despite the observed decline in cellular viability, no evident histopathological alterations were detected in the hepatopancreas. This apparent resistance may be related to the organ’s high metabolic activity and its key role in the detoxification of ROS and xenobiotics [83].

These results are consistent with the histopathological observations, which showed limited alterations in muscle tissue but evident structural changes in gill tissues, particularly after prolonged exposure. The occurrence of epithelial remodeling in the gills suggests that this organ represents a primary site of physiological adjustment to PVA exposure, likely reflecting its role as a key interface between the organism and the surrounding environment. A more quantitative histopathological approach may provide additional insight into PVA effects, enabling a more comprehensive evaluation at both cellular and tissue levels.

Overall, both the available literature and the present findings indicate that oxidative stress biomarkers provide significant advantages over classical ecotoxicological tests, as they allow for the detection of early biological effects, the interpretation of specific mechanisms of action, and the monitoring of sublethal responses before impacts become evident at the individual or population level. Consequently, these approaches represent more sensitive and predictive tools for assessing the environmental impact of emerging contaminants [107]. Although this represents the first application of this integrative approach in *C. sapidus*, the results appear promising and complement the information obtained from the biomarker panel, highlighting the usefulness of multibiomarker strategies for evaluating sublethal stress responses in marine crustaceans.

Finally, using invasive species such as *C. sapidus* as experimental models in ecotoxicological studies offers several advantages. The use of this species reduces the need to sample native fauna, thereby limiting additional pressure on local ecosystems [108]. Moreover, *C. sapidus* is widely distributed across different geographic regions, which allows for comparisons of physiological and toxicological responses among ecosystems with differing environmental conditions [109]. Although not applied here as a biomonitoring tool, the employment of *C. sapidus* as a laboratory model is consistent with population management strategies, as it involves the removal of individuals from invaded habitats while simultaneously providing ecologically relevant information on the biological effects of emerging contaminants [49,53,110,111,112,113,114,115].

## 5. Conclusions

PVA exposure did not induce mortality in *Callinectes sapidus*, confirming its low acute toxicity under controlled conditions. However, clear sublethal effects were observed, including reduced cell viability, tissue-specific adaptive responses, and significant oxidative stress. The hepatopancreas and gills emerged as the most sensitive organs, showing both biochemical and structural alterations, while muscle tissue responded more slowly. Biomarker integration (IBR-T and NMDS) highlighted a consistent dose- and time-dependent physiological disturbance. Overall, these findings demonstrate that PVA, despite being considered a low toxicity polymer, can impair physiological homeostasis under prolonged exposure, emphasizing the importance of multibiomarker approaches for detecting early ecological risks of emerging contaminants.

Future research should focus on long-term and environmentally realistic exposures, including mixture scenarios with other contaminants, to better reflect natural conditions. This should include monitoring of PVA concentrations in water and measurements of bioaccumulation in organisms.

Further investigation into the interaction of PVA with antioxidant and detoxification pathways, particularly the glutathione system, is needed to clarify its mechanisms of action. In addition, expanding studies to different life stages and species, as well as integrating molecular and omics approaches, would improve the understanding of ecological consequences and support more accurate environmental risk assessments.

Finally, the use of *Callinectes sapidus* as a model organism demonstrates how ecotoxicological research on emerging contaminants can be effectively integrated with management strategies aimed at controlling invasive species, thereby linking environmental risk assessment with ecosystem-level mitigation practices.

## Figures and Tables

**Table 1:** Mean and standard deviation of the physicochemical water parameters (n = 20 for each parameter) recorded during the exposure of *Callinectes sapidus* to PVA (CTRL: control; C1: 0.5 mg L^−1^; C2: 5 mg L^−1^; C3: 25 mg L^−1^).

Experimental Group	Temperature (°C)	pH	Salinity (psu)	Oxygen (ppm)
CTRL	23.59 ± 2.34	8.04 ± 0.23	17.08 ± 0.61	6.22 ± 0.71
C1	24.18 ± 2.46	8.03 ± 0.16	16.93 ± 0.51	6.35 ± 0.68
C2	24.07 ± 2.67	8.01 ± 0.32	16.96 ± 0.50	5.77 ± 1.04
C3	24.15 ± 2.70	8.01 ± 0.24	16.98 ± 0.65	5.74 ± 1.36

**Table 2:** Mean and standard deviation of weight (g) and carapax length (cm) of *Callinectes sapidus* to PVA exposure treatments (CTRL: control; C1: 0.5 mg L^−1^; C2: 5 mg L^−1^; C3: 25 mg L^−1^).

Biometric Feature	CTRL	C1	C2	C3
Weight (g)	23.59 ± 2.34	24.18 ± 2.46	24.07 ± 2.67	24.15 ± 2.70
Carapax length (cm)	8.04 ± 0.23	8.10 ± 0.16	7.99 ± 0.32	8.01 ± 0.24

## Data Availability

The original contributions presented in this study are included in this article/Supplementary Materials. Further inquiries can be directed to the corresponding authors.

## Supplementary Materials

The following supporting information can be downloaded at: https://www.mdpi.com/article/10.3390/toxics14050358/s1, Figure S1: Boxplot of (a) weight (g) and (b) carapax length (cm) of *Callinectes sapidus* to PVA exposure treatments (CTRL: control; C1: 0.5 mg L^−1^; C2: 5 mg L^−1^; C3: 25 mg L^−1^). Kruskal–Wallis test showed no significant differences among the different treatment groups (*p* > 0.05); Figure S2: Number of individuals by sex (F = females, M = males) across the different treatment groups (CTRL: control; C1: 0.5 mg L^−1^; C2: 5 mg L^−1^; C3: 25 mg L^−1^). Values above the bars indicate the number of individuals in each group; Table S1: Percentage of the hemolymph (HE) and hepatopancreas (HP) cell viability in *Callinectes sapidus* (n = 4 per treatment) after 10 and 20 days of exposure to PVA at different concentrations (CTRL: control; C1: 0.5 mg L^−1^; C2: 5 mg L^−1^; C3: 25 mg L^−1^), following Trypan Blue (TB) exclusion method and the Neutral Red (NR) retention assay. Cell viability is expressed in percentage (%) as mean ± standard deviation; Figure S3: A comparison has been made between viable and non-viable cells in the neutral red retention test (NR) and the trypan blue exclusion test (TB). The images show: (a) viable cell in NR; (b) non-viable cell in NR; (c) viable cell in TB; (d) non-viable cell in TB; Table S2: Integrative Biomarker Response-Threshold (IBR-T) values calculated for gills (G), hepatopancreas (HP), and muscle (M) of *Callinectes sapidus* after 10 days of exposure to PVA (CTRL: control; C1: 0.5 mg L^−1^; C2: 5 mg L^−1^; C3: 25 mg L^−1^); Table S3: Integrative Biomarker Response-Threshold (IBR-T) values calculated for gills (G), hepatopancreas (HP), and muscle (M) of *Callinectes sapidus* after 20 days of exposure to PVA (CTRL: control; C1: 0.5 mg L^−1^; C2: 5 mg L^−1^; C3: 25 mg L^−1^).
