# Supplementary material for: The Invasive Blue Crab Callinectes sapidus as a Model for Assessing Sub-Lethal Effects of Polyvinyl Alcohol
Source: Toxics. 2026 Apr 24;14(5):358. doi: 10.3390/toxics14050358 (PMC13211180; doi:10.3390/toxics14050358)
Supplement: Supplementary file 1 [file toxics-14-00358-s001.zip › toxics-4245647-supplementary.docx]

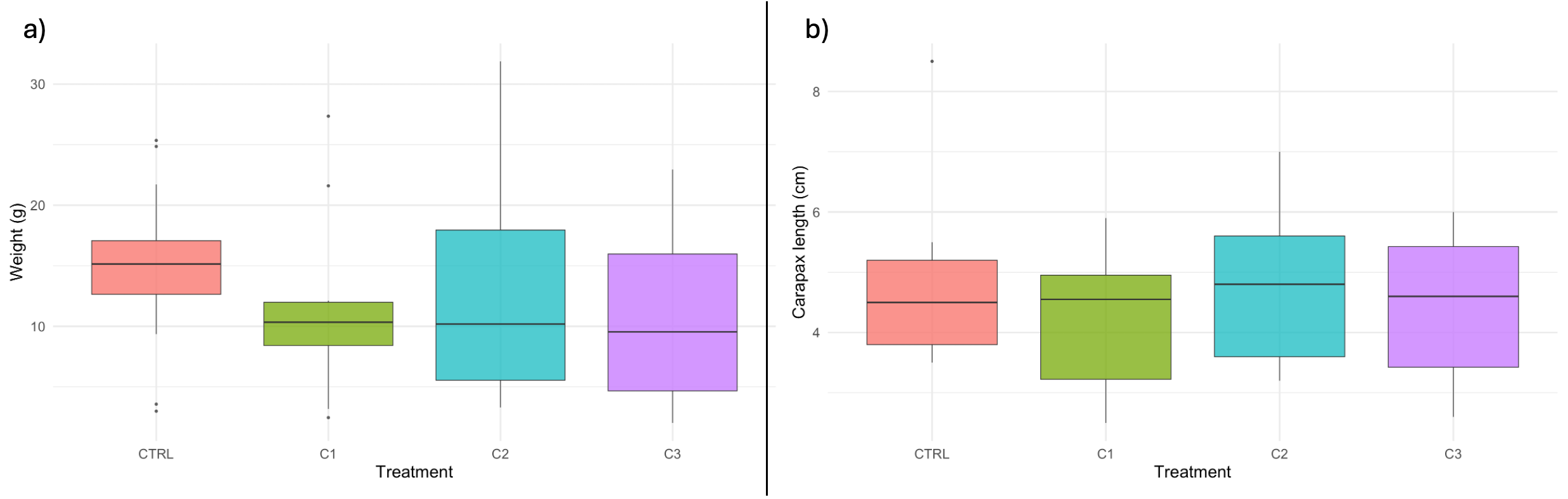
**Figure S1**. Boxplot of a) weight (g) and b) carapax length (cm) of *Callinectes sapidus* to PVA exposure treatments (CTRL: control; C1: 0.5 mg L⁻¹; C2: 5 mg L⁻¹; C3: 25 mg L⁻¹). Kruskal–Wallis test showed no significant differences among the different treatment groups (*p* > 0.05).


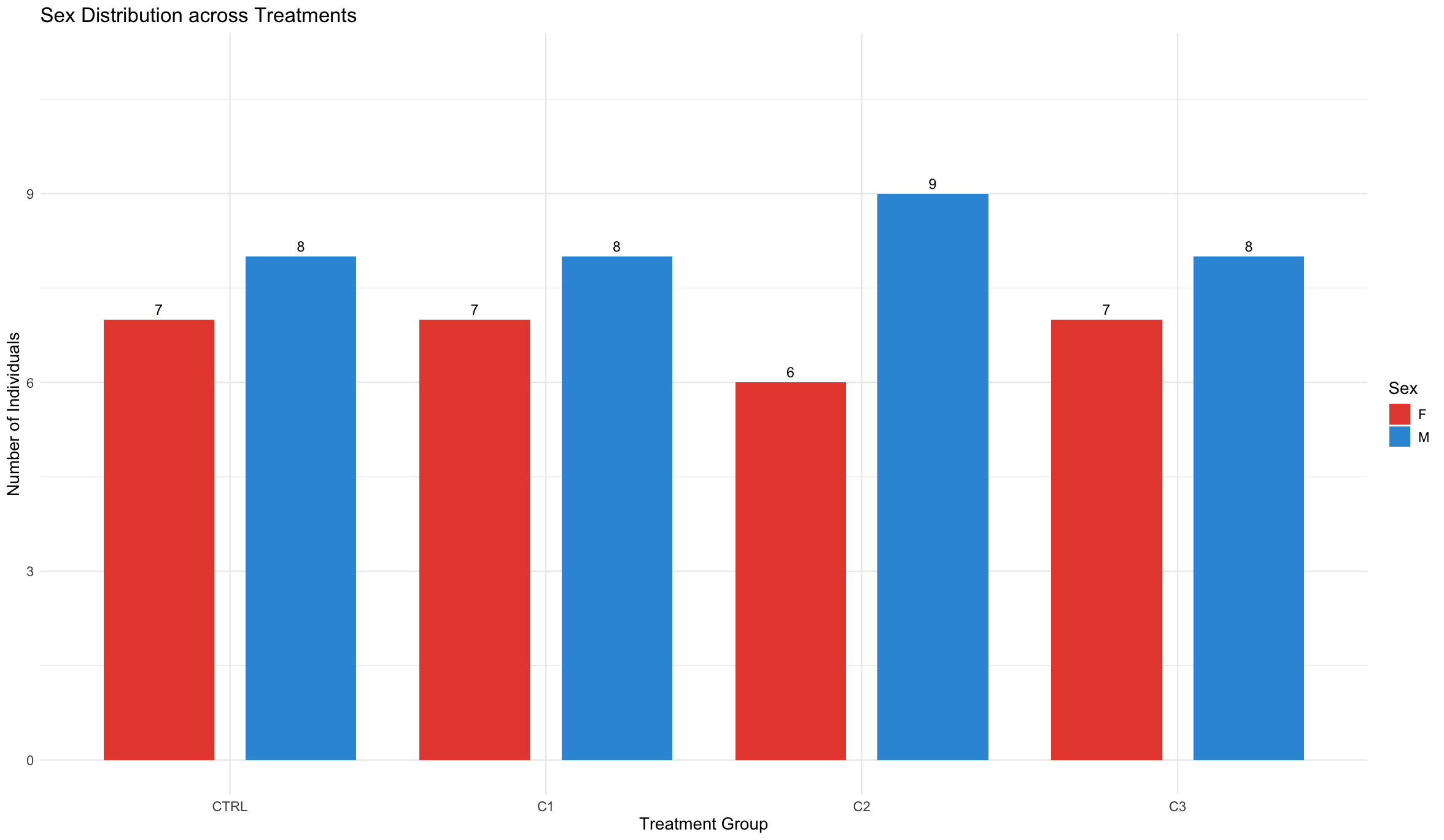


**Figure S2**. Number of individuals by sex (F = females, M = males) across the different treatment groups (CTRL: control; C1: 0.5 mg L⁻¹; C2: 5 mg L⁻¹; C3: 25 mg L⁻¹). Values above the bars indicate the number of individuals in each group.

**Table S1.** Percentage of the haemolymph (HE) and hepatopancreas (HP) cell viability in *Callinectes sapidus* (n = 4 per treatment) after 10 and 20 days of exposure to PVA at different concentrations (CTRL: control; C1: 0.5 mg L⁻¹; C2: 5 mg L⁻¹; C3: 25 mg L⁻¹). following Trypan Blue (TB) exclusion method and the Neutral Red (NR) retention assay. Cell viability is expressed in percentage (%) as mean ± standard deviation.

| **Days of exposure** | **Method** | **Matrix** | **Treatments** | | | |
| --- | --- | --- | --- | --- | --- | --- |
|  |  |  | **CTRL** | **C1** | **C2** | **C3** |
| 10 | TB | HE | 99.43 ± 0.23 | 99.67 ± 0.27 | 99.35 ± 0.30 | 94.24 ± 2.83 |
|  | NR | HE | 99.40 ± 0.77 | 99.84 ± 0.25 | 99.78 ± 0.31 | 99.62 ± 0.30 |
|  | TB | HP | 99.77 ± 0.36 | 96.33 ± 1.71 | 98.95 ± 1.80 | 94.48 ± 1.87 |
|  | NR | HP | 96.99 ± 1.21 | 97.21 ± 3.24 | 94.41 ± 3.97 | 85.46 ± 5.84 |
| 20 | TB | HE | 99.88 ± 0.09 | 99.52 ± 0.32 | 90.83 ± 3.52 | 92.05 ± 3.77 |
|  | NR | HE | 99.86 ± 0.11 | 99.76 ± 0.33 | 99.87 ± 0.09 | 99.31 ± 0.32 |
|  | TB | HP | 100 ± 0 | 96.07 ± 0.67 | 100 ± 0 | 94.50 ± 2.29 |
|  | NR | HP | 98.32 ± 0.62 | 90.82 ± 2.07 | 69.90 ± 9.30 | 79.90 ± 4.83 |


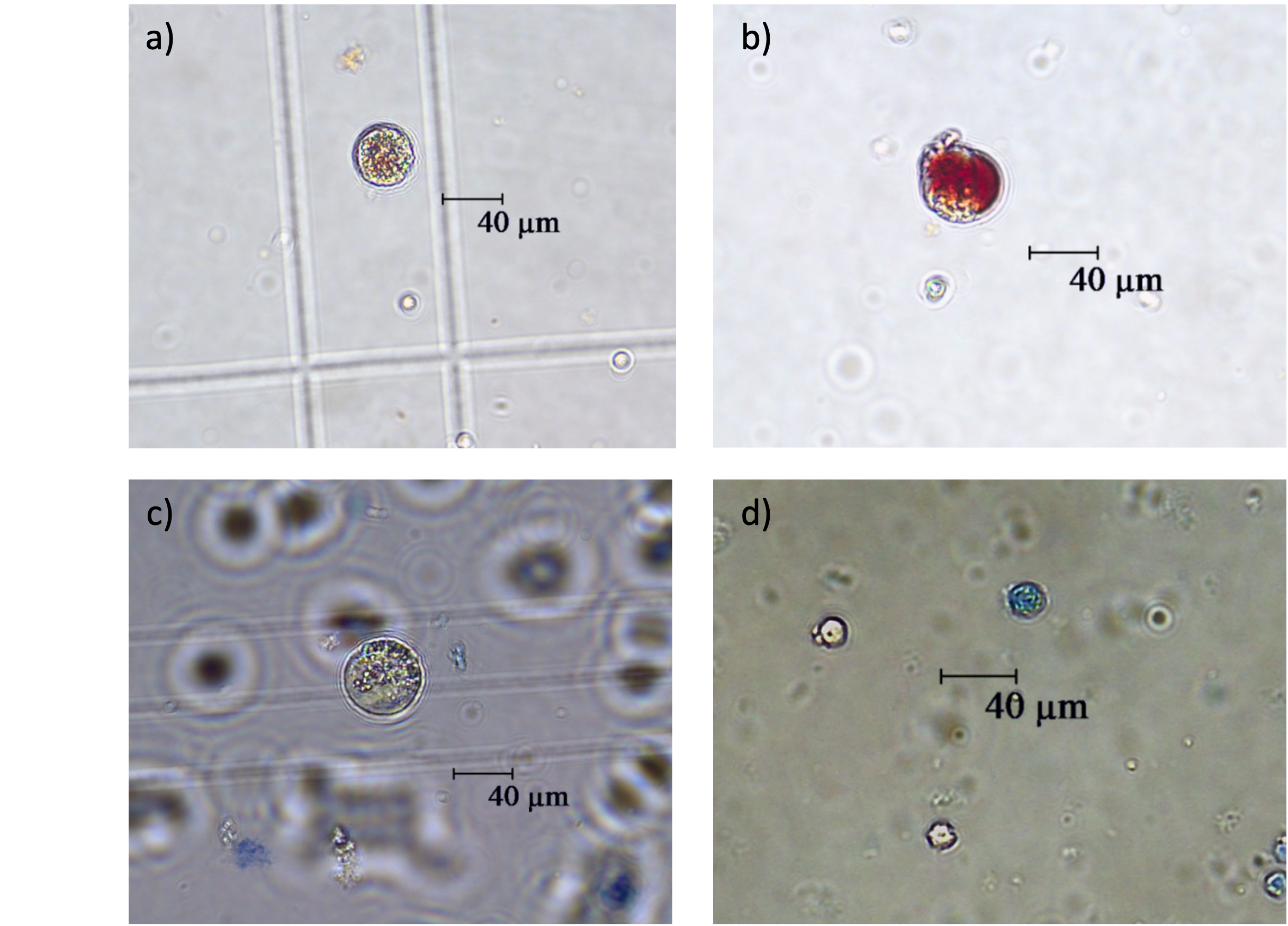
**Figure S3**. A comparison has been made between viable and non-viable cells in the neutral red retention test (NR) and the trypan blue exclusion test (TB). The images show: (a) viable cell in NR; (b) non-viable cell in NR; (c) viable cell in TB; (d) non-viable cell in TB.

**Table S2**. Integrative Biomarker Response-Threshold (IBR-T) values calculated for gills (G). hepatopancreas (HP). and muscle (M) of *Callinectes sapidus* after 10 days of exposure to PVA (CTRL: control; C1: 0.5 mg L⁻¹; C2: 5 mg L⁻¹; C3: 25 mg L⁻¹).

| Tissue | Treatment | Biomarker | RawValue | Reference | log_xx0 | A_jk | IBRT |
| --- | --- | --- | --- | --- | --- | --- | --- |
| G | C1 | GPx | 10.0217 | 10.5842 | -0.0237 | -0.3168 | 0.5909 |
| G | C1 | GST | 0.1426 | 0.1578 | -0.0441 | -0.8870 | 0.5909 |
| G | C1 | LPO | 0.9125 | 0.5875 | 0.1912 | 0.7826 | 0.5909 |
| G | C1 | SOD | 2.7517 | 2.5375 | 0.0352 | 0.3773 | 0.5909 |
| G | C2 | GPx | 11.9933 | 10.5842 | 0.0543 | 0.7250 | 1.0559 |
| G | C2 | GST | 0.1696 | 0.1578 | 0.0312 | 0.6268 | 1.0559 |
| G | C2 | LPO | 1.2267 | 0.5875 | 0.3197 | 1.3085 | 1.0559 |
| G | C2 | SOD | 3.5500 | 2.5375 | 0.1458 | 1.5635 | 1.0559 |
| G | C3 | GPx | 7.9450 | 10.5842 | -0.1246 | -1.6637 | 1.9600 |
| G | C3 | GST | 0.1306 | 0.1578 | -0.0823 | -1.6543 | 1.9600 |
| G | C3 | LPO | 2.2458 | 0.5875 | 0.5824 | 2.3834 | 1.9600 |
| G | C3 | SOD | 4.0167 | 2.5375 | 0.1995 | 2.1386 | 1.9600 |
| G | CTRL | GPx | 10.5842 | 10.5842 | 0 | 0 | 0 |
| G | CTRL | GST | 0.1578 | 0.1578 | 0 | 0 | 0 |
| G | CTRL | LPO | 0.5875 | 0.5875 | 0 | 0 | 0 |
| G | CTRL | SOD | 2.5375 | 2.5375 | 0 | 0 | 0 |
| HP | C1 | GPx | 12.3142 | 10.5308 | 0.0679 | 1.1496 | 0.7464 |
| HP | C1 | GST | 0.1079 | 0.1554 | -0.1584 | -1.0972 | 0.7464 |
| HP | C1 | LPO | 0.9800 | 0.6717 | 0.1641 | 0.7338 | 0.7464 |
| HP | C1 | SOD | 2.3833 | 2.3808 | 0.0005 | 0.0047 | 0.7464 |
| HP | C2 | GPx | 9.0975 | 10.5308 | -0.0635 | -1.0751 | 0.8660 |
| HP | C2 | GST | 0.1902 | 0.1554 | 0.0876 | 0.6070 | 0.8660 |
| HP | C2 | LPO | 1.0008 | 0.6717 | 0.1732 | 0.7747 | 0.8660 |
| HP | C2 | SOD | 2.9750 | 2.3808 | 0.0968 | 1.0072 | 0.8660 |
| HP | C3 | GPx | 9.4300 | 10.5308 | -0.0480 | -0.8113 | 1.6341 |
| HP | C3 | GST | 0.2358 | 0.1554 | 0.1811 | 1.2544 | 1.6341 |
| HP | C3 | LPO | 2.2775 | 0.6717 | 0.5303 | 2.3718 | 1.6341 |
| HP | C3 | SOD | 3.7875 | 2.3808 | 0.2016 | 2.0987 | 1.6341 |
| HP | CTRL | GPx | 10.5308 | 10.5308 | 0 | 0 | 0 |
| HP | CTRL | GST | 0.1554 | 0.1554 | 0 | 0 | 0 |
| HP | CTRL | LPO | 0.6717 | 0.6717 | 0 | 0 | 0 |
| HP | CTRL | SOD | 2.3808 | 2.3808 | 0 | 0 | 0 |
| M | C1 | GPx | 9.7408 | 10.5842 | -0.0361 | -1.6417 | 0.7978 |
| M | C1 | GST | 0.1464 | 0.1787 | -0.0865 | -0.6646 | 0.7978 |
| M | C1 | LPO | 0.8475 | 0.5333 | 0.2011 | 0.7666 | 0.7978 |
| M | C1 | SOD | 2.5417 | 2.4800 | 0.0107 | 0.1183 | 0.7978 |
| M | C2 | GPx | 10.9458 | 10.5842 | 0.0146 | 0.6643 | 0.9020 |
| M | C2 | GST | 0.1953 | 0.1787 | 0.0385 | 0.2963 | 0.9020 |
| M | C2 | LPO | 1.3142 | 0.5333 | 0.3917 | 1.4927 | 0.9020 |
| M | C2 | SOD | 3.1517 | 2.4800 | 0.1041 | 1.1546 | 0.9020 |
| M | C3 | GPx | 10.1550 | 10.5842 | -0.0180 | -0.8184 | 1.7500 |
| M | C3 | GST | 0.2981 | 0.1787 | 0.2223 | 1.7087 | 1.7500 |
| M | C3 | LPO | 2.1908 | 0.5333 | 0.6136 | 2.3387 | 1.7500 |
| M | C3 | SOD | 3.8625 | 2.4800 | 0.1924 | 2.1343 | 1.7500 |
| M | CTRL | GPx | 10.5842 | 10.5842 | 0 | 0 | 0 |
| M | CTRL | GST | 0.1787 | 0.1787 | 0 | 0 | 0 |
| M | CTRL | LPO | 0.5333 | 0.5333 | 0 | 0 | 0 |
| M | CTRL | SOD | 2.4800 | 2.4800 | 0 | 0 | 0 |

**Table S3**. Integrative Biomarker Response-Threshold (IBR-T) values calculated for gills (G). hepatopancreas (HP). and muscle (M) of *Callinectes sapidus* after 20 days of exposure to PVA (CTRL: control; C1: 0.5 mg L⁻¹; C2: 5 mg L⁻¹; C3: 25 mg L⁻¹).

| Tissue | Treatment | Biomarker | RawValue | Reference | log_xx0 | A_jk | IBRT |
| --- | --- | --- | --- | --- | --- | --- | --- |
| G | C1 | GPx | 12.9613 | 9.2671 | 0.1457 | 2.0712 | 1.3018 |
| G | C1 | GST | 0.1418 | 0.2039 | -0.1580 | -1.8402 | 1.3018 |
| G | C1 | LPO | 0.8600 | 0.7536 | 0.0574 | 0.2951 | 1.3018 |
| G | C1 | SOD | 2.9525 | 2.3443 | 0.1002 | 1.0009 | 1.3018 |
| G | C2 | GPx | 10.2207 | 9.2671 | 0.0425 | 0.6047 | 0.8378 |
| G | C2 | GST | 0.1974 | 0.2039 | -0.0141 | -0.1639 | 0.8378 |
| G | C2 | LPO | 1.2121 | 0.7536 | 0.2064 | 1.0617 | 0.8378 |
| G | C2 | SOD | 3.3286 | 2.3443 | 0.1522 | 1.5211 | 0.8378 |
| G | C3 | GPx | 9.1250 | 9.2671 | -0.0067 | -0.0954 | 1.2928 |
| G | C3 | GST | 0.2226 | 0.2039 | 0.0380 | 0.4429 | 1.2928 |
| G | C3 | LPO | 2.0550 | 0.7536 | 0.4357 | 2.2409 | 1.2928 |
| G | C3 | SOD | 4.0683 | 2.3443 | 0.2394 | 2.3918 | 1.2928 |
| G | CTRL | GPx | 9.2671 | 9.2671 | 0 | 0 | 0 |
| G | CTRL | GST | 0.2039 | 0.2039 | 0 | 0 | 0 |
| G | CTRL | LPO | 0.7536 | 0.7536 | 0 | 0 | 0 |
| G | CTRL | SOD | 2.3443 | 2.3443 | 0 | 0 | 0 |
| HP | C1 | GPx | 13.5650 | 11.3107 | 0.0789 | 1.8636 | 1.0173 |
| HP | C1 | GST | 0.1949 | 0.1362 | 0.1555 | 1.5857 | 1.0173 |
| HP | C1 | LPO | 0.9350 | 0.6657 | 0.1475 | 0.5944 | 1.0173 |
| HP | C1 | SOD | 2.4463 | 2.4586 | -0.0022 | -0.0254 | 1.0173 |
| HP | C2 | GPx | 11.7000 | 11.3107 | 0.0147 | 0.3470 | 0.8944 |
| HP | C2 | GST | 0.1887 | 0.1362 | 0.1416 | 1.4434 | 0.8944 |
| HP | C2 | LPO | 0.9429 | 0.6657 | 0.1512 | 0.6091 | 0.8944 |
| HP | C2 | SOD | 3.1050 | 2.4586 | 0.1014 | 1.1783 | 0.8944 |
| HP | C3 | GPx | 10.8375 | 11.3107 | -0.0186 | -0.4382 | 1.8004 |
| HP | C3 | GST | 0.2345 | 0.1362 | 0.2359 | 2.4052 | 1.8004 |
| HP | C3 | LPO | 2.5050 | 0.6657 | 0.5755 | 2.3190 | 1.8004 |
| HP | C3 | SOD | 3.6825 | 2.4586 | 0.1755 | 2.0392 | 1.8004 |
| HP | CTRL | GPx | 11.3107 | 11.3107 | 0 | 0 | 0 |
| HP | CTRL | GST | 0.1362 | 0.1362 | 0 | 0 | 0 |
| HP | CTRL | LPO | 0.6657 | 0.6657 | 0 | 0 | 0 |
| HP | CTRL | SOD | 2.4586 | 2.4586 | 0 | 0 | 0 |
| M | C1 | GPx | 12.5788 | 8.6914 | 0.1605 | 1.8619 | 0.8912 |
| M | C1 | GST | 0.1570 | 0.1273 | 0.0911 | 0.7494 | 0.8912 |
| M | C1 | LPO | 0.8750 | 0.6414 | 0.1349 | 0.5354 | 0.8912 |
| M | C1 | SOD | 2.5363 | 2.3221 | 0.0383 | 0.4179 | 0.8912 |
| M | C2 | GPx | 12.0121 | 8.6914 | 0.1405 | 1.6298 | 1.1028 |
| M | C2 | GST | 0.1468 | 0.1273 | 0.0619 | 0.5091 | 1.1028 |
| M | C2 | LPO | 1.2071 | 0.6414 | 0.2746 | 1.0902 | 1.1028 |
| M | C2 | SOD | 2.9800 | 2.3221 | 0.1083 | 1.1820 | 1.1028 |
| M | C3 | GPx | 13.6783 | 8.6914 | 0.1969 | 2.2840 | 2.3034 |
| M | C3 | GST | 0.2437 | 0.1273 | 0.2820 | 2.3194 | 2.3034 |
| M | C3 | LPO | 2.4817 | 0.6414 | 0.5876 | 2.3328 | 2.3034 |
| M | C3 | SOD | 3.7550 | 2.3221 | 0.2087 | 2.2774 | 2.3034 |
| M | CTRL | GPx | 8.6914 | 8.6914 | 0 | 0 | 0 |
| M | CTRL | GST | 0.1273 | 0.1273 | 0 | 0 | 0 |
| M | CTRL | LPO | 0.6414 | 0.6414 | 0 | 0 | 0 |
| M | CTRL | SOD | 2.3221 | 2.3221 | 0 | 0 | 0 |
